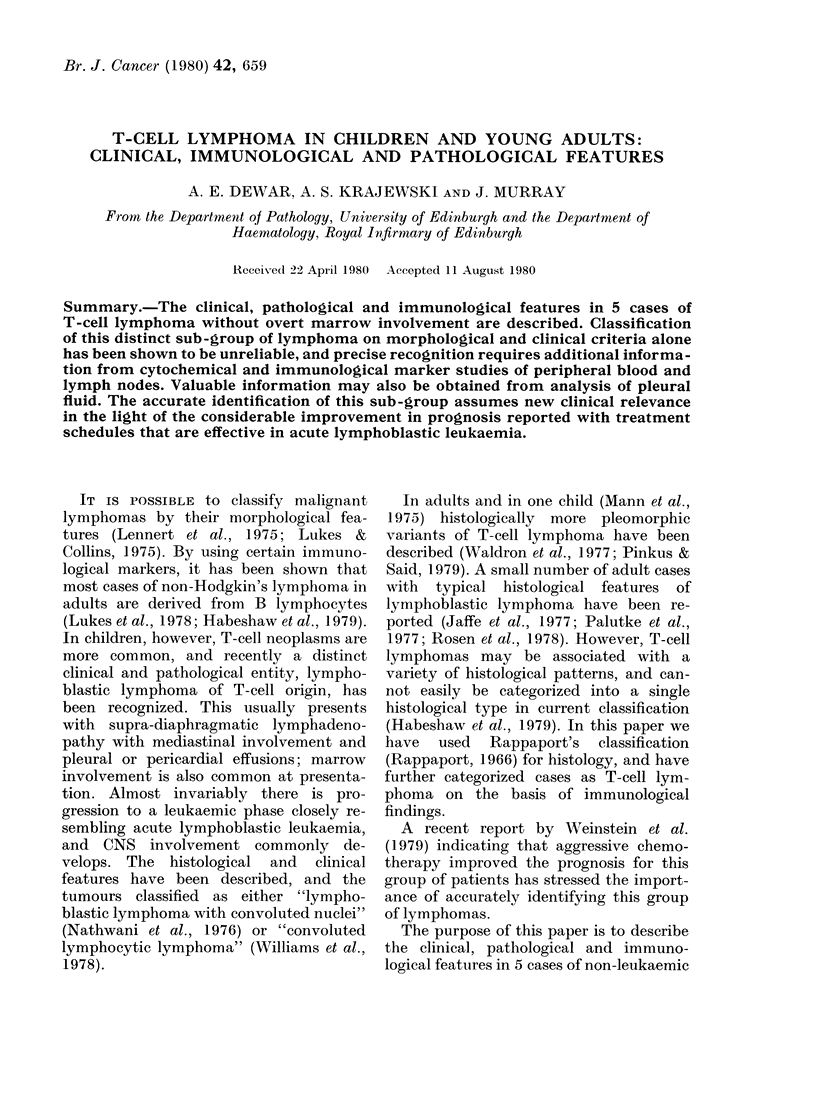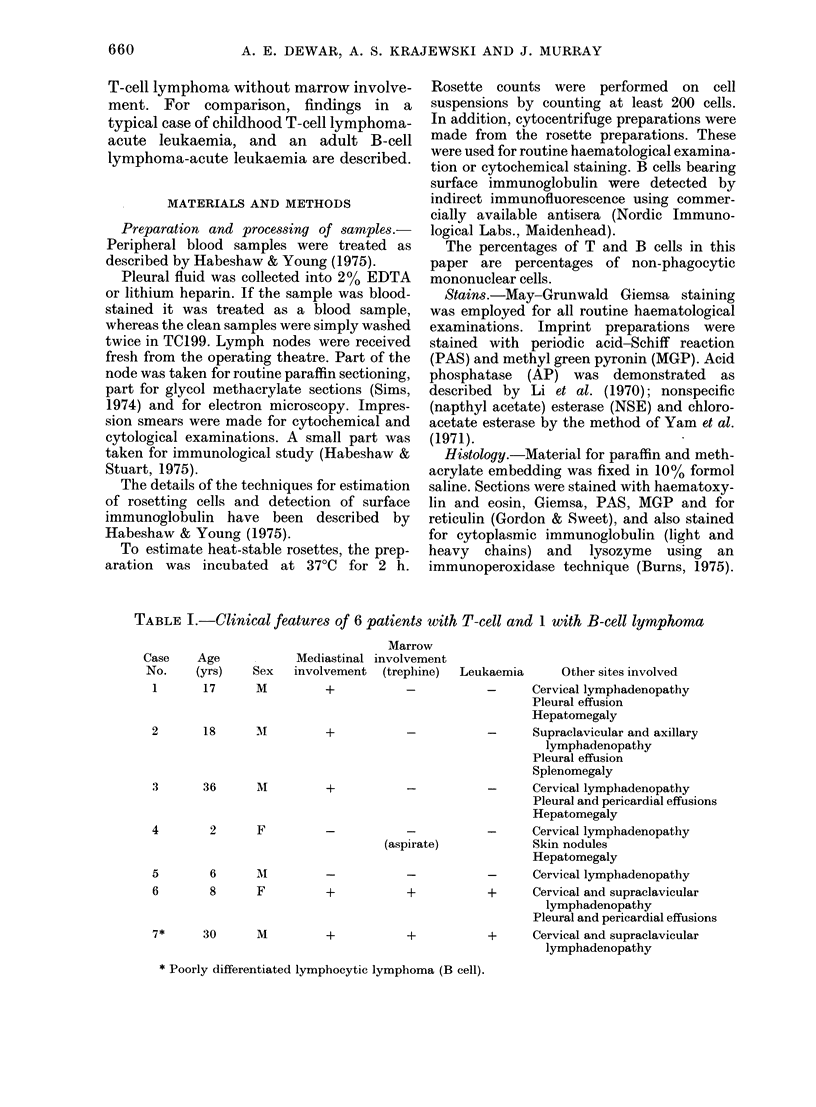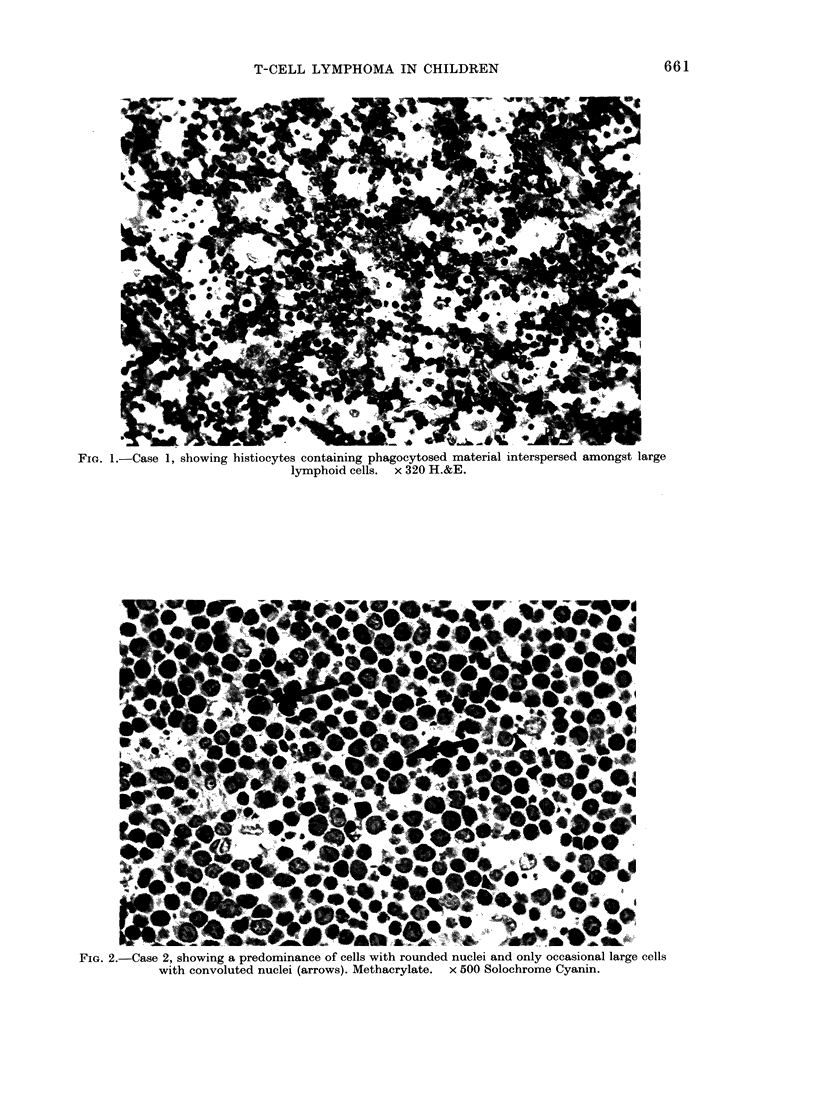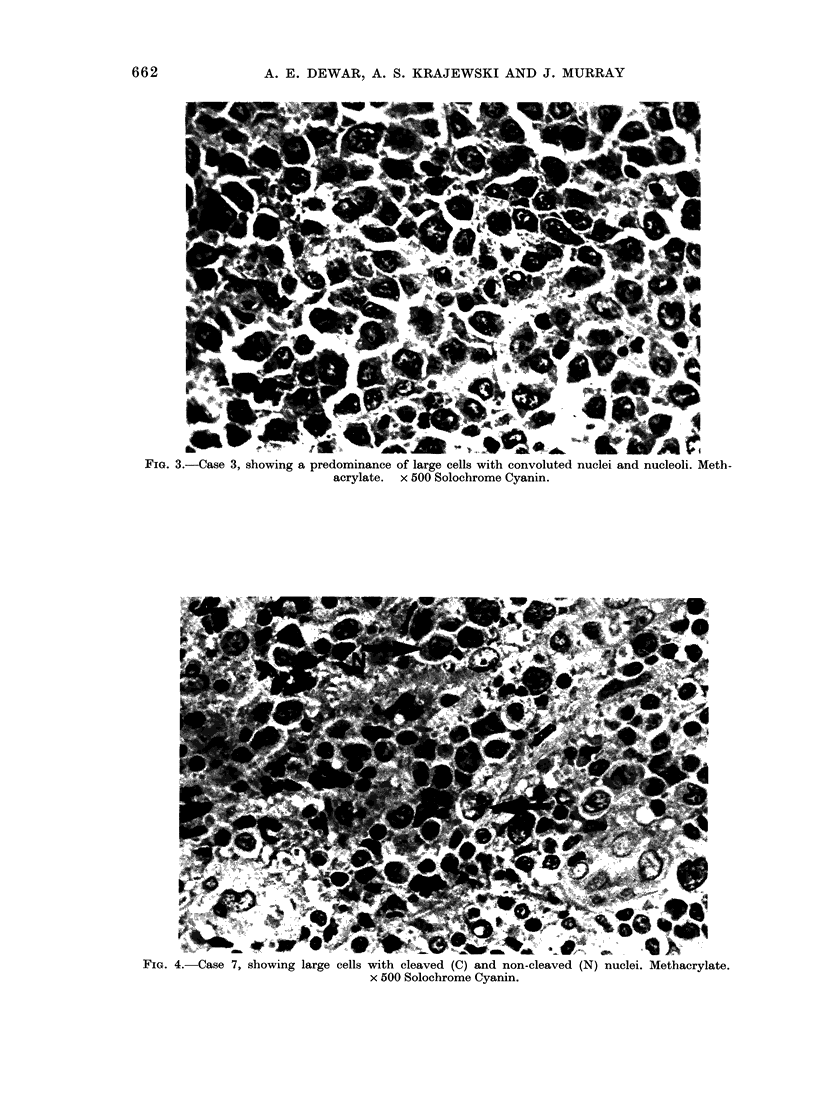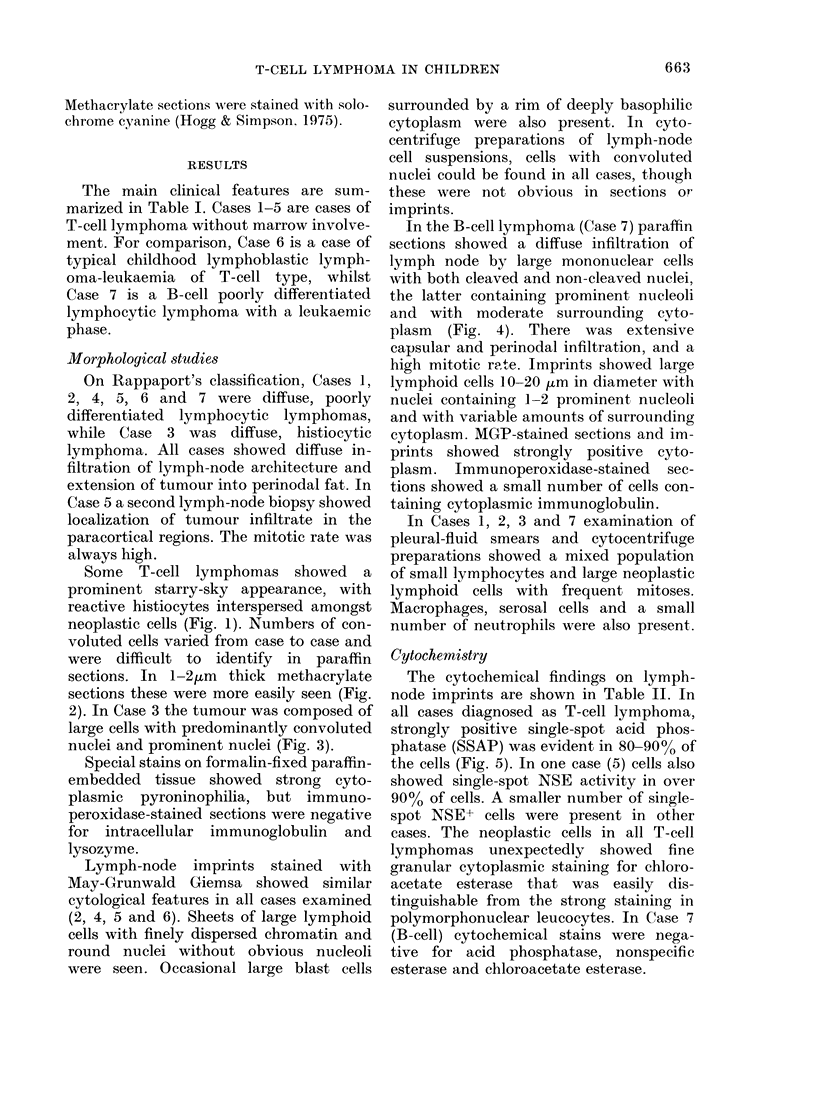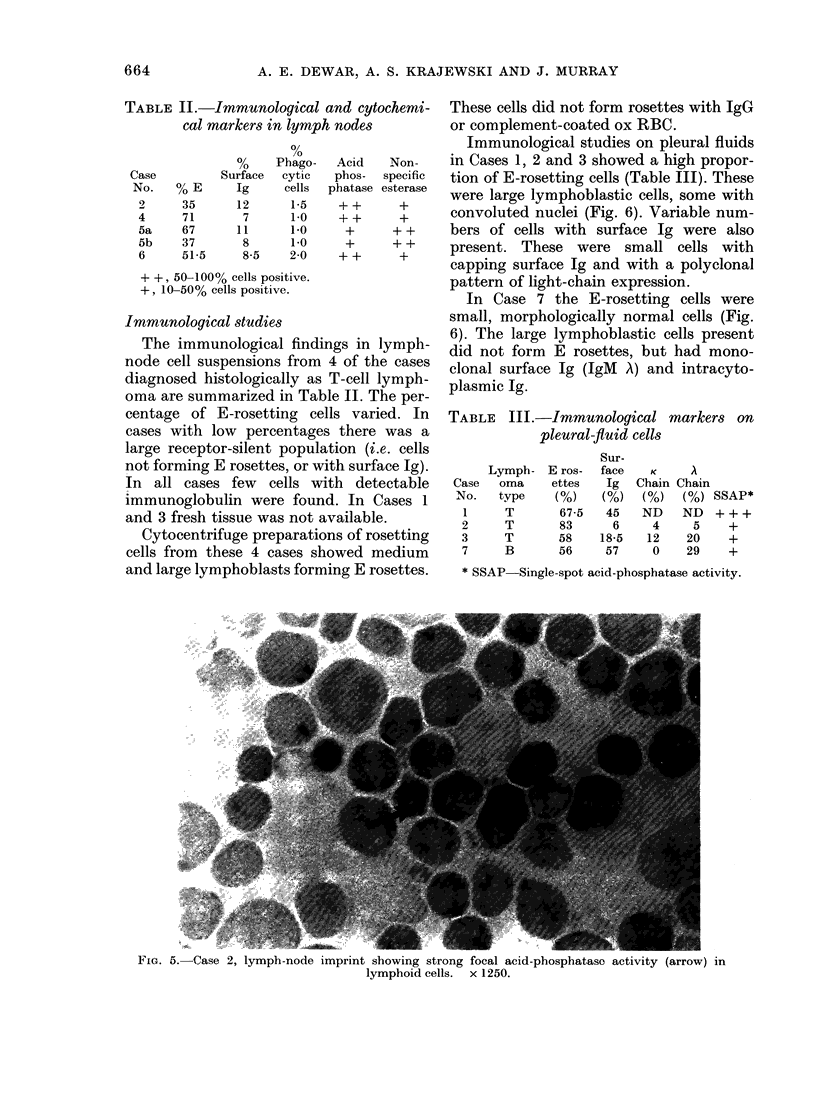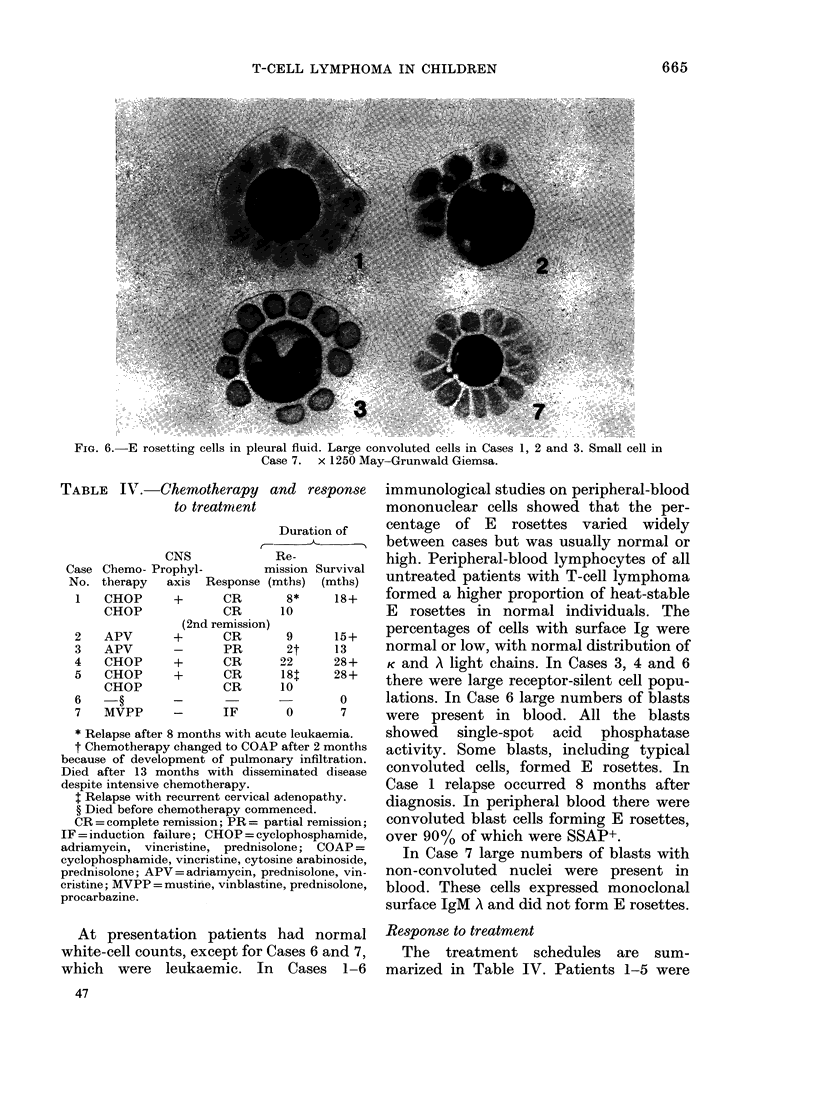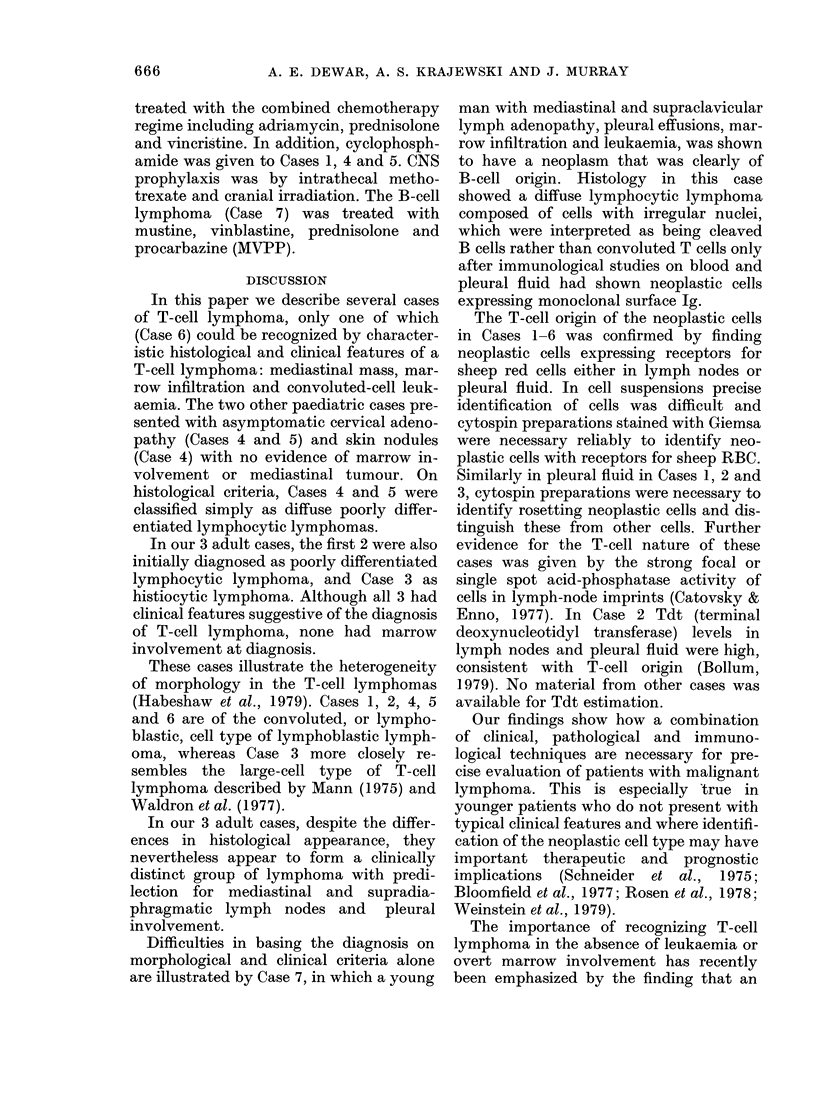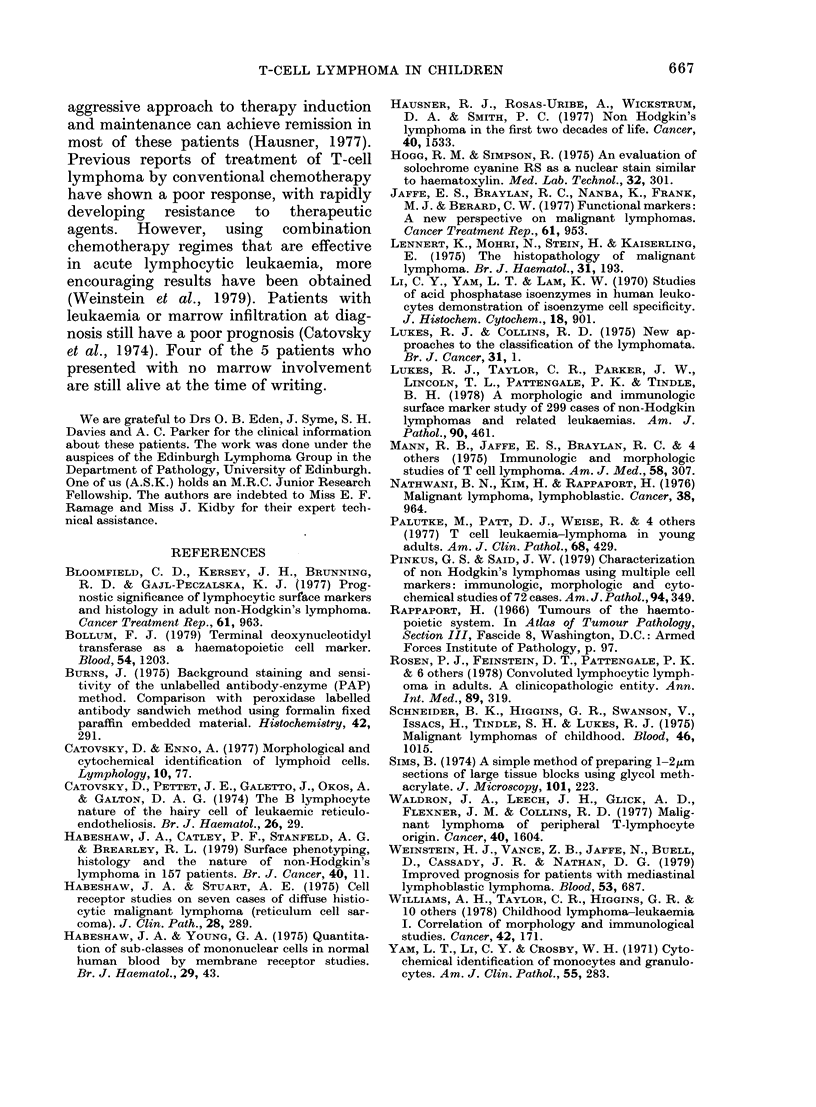# T-cell lymphoma in children and young adults: clinical, immunological and pathological features.

**DOI:** 10.1038/bjc.1980.299

**Published:** 1980-11

**Authors:** A. E. Dewar, A. S. Krajewski, J. Murray

## Abstract

**Images:**


*Br. J. Cancer* (1980) **42**, 659

### T-CELL LYMPHOMA IN CHILDREN AND YOUNG ADULTS: CLINICAL, IMMUNOLOGICAL AND PATHOLOGICAL FEATURES

A. E. DEWAR, A. S. KRAJEWSKI AND J. MURRAY

*From the Department of Pathology, University of Edinburgh and the Department of Haematology, Royal Infirmary of Edinburgh*

Received 22 April 1980 Accepted 11 August 1980

**Summary.—The clinical, pathological and immunological features in 5 cases of T-cell lymphoma without overt marrow involvement are described. Classification of this distinct sub-group of lymphoma on morphological and clinical criteria alone has been shown to be unreliable, and precise recognition requires additional information from cytochemical and immunological marker studies of peripheral blood and lymph nodes. Valuable information may also be obtained from analysis of pleural fluid. The accurate identification of this sub-group assumes new clinical relevance in the light of the considerable improvement in prognosis reported with treatment schedules that are effective in acute lymphoblastic leukaemia.**

IT IS POSSIBLE to classify malignant lymphomas by their morphological features (Lennert *et al.*, 1975; Lukes & Collins, 1975). By using certain immunological markers, it has been shown that most cases of non-Hodgkin's lymphoma in adults are derived from B lymphocytes (Lukes *et al.*, 1978; Habeshaw *et al.*, 1979). In children, however, T-cell neoplasms are more common, and recently a distinct clinical and pathological entity, lymphoblastic lymphoma of T-cell origin, has been recognized. This usually presents with supra-diaphragmatic lymphadenopathy with mediastinal involvement and pleural or pericardial effusions; marrow involvement is also common at presentation. Almost invariably there is progression to a leukaemic phase closely resembling acute lymphoblastic leukaemia, and CNS involvement commonly develops. The histological and clinical features have been described, and the tumours classified as either "lymphoblastic lymphoma with convoluted nuclei" (Nathwani *et al.*, 1976) or "convoluted lymphocytic lymphoma" (Williams *et al.*, 1978).

In adults and in one child (Mann *et al.*, 1975) histologically more pleomorphic variants of T-cell lymphoma have been described (Waldron *et al.*, 1977; Pinkus & Said, 1979). A small number of adult cases with typical histological features of lymphoblastic lymphoma have been reported (Jaffe *et al.*, 1977; Palutke *et al.*, 1977; Rosen *et al.*, 1978). However, T-cell lymphomas may be associated with a variety of histological patterns, and cannot easily be categorized into a single histological type in current classification (Habeshaw *et al.*, 1979). In this paper we have used Rappaport's classification (Rappaport, 1966) for histology, and have further categorized cases as T-cell lymphoma on the basis of immunological findings.

A recent report by Weinstein *et al.* (1979) indicating that aggressive chemotherapy improved the prognosis for this group of patients has stressed the importance of accurately identifying this group of lymphomas.

The purpose of this paper is to describe the clinical, pathological and immunological features in 5 cases of non-leukaemic

T-cell lymphoma without marrow involvement. For comparison, findings in a typical case of childhood T-cell lymphoma-acute leukaemia, and an adult B-cell lymphoma-acute leukaemia are described.

### MATERIALS AND METHODS

*Preparation and processing of samples.*—Peripheral blood samples were treated as described by Habeshaw & Young (1975).

Pleural fluid was collected into 2% EDTA or lithium heparin. If the sample was blood-stained it was treated as a blood sample, whereas the clean samples were simply washed twice in TC199. Lymph nodes were received fresh from the operating theatre. Part of the node was taken for routine paraffin sectioning, part for glycol methacrylate sections (Sims, 1974) and for electron microscopy. Impression smears were made for cytochemical and cytological examinations. A small part was taken for immunological study (Habeshaw & Stuart, 1975).

The details of the techniques for estimation of rosetting cells and detection of surface immunoglobulin have been described by Habeshaw & Young (1975).

To estimate heat-stable rosettes, the preparation was incubated at 37°C for 2 h. Rosette counts were performed on cell suspensions by counting at least 200 cells. In addition, cytocentrifuge preparations were made from the rosette preparations. These were used for routine haematological examination or cytochemical staining. B cells bearing surface immunoglobulin were detected by indirect immunofluorescence using commercially available antisera (Nordic Immunological Labs., Maidenhead).

The percentages of T and B cells in this paper are percentages of non-phagocytic mononuclear cells.

*Stains.*—May–Grunwald Giemsa staining was employed for all routine haematological examinations. Imprint preparations were stained with periodic acid–Schiff reaction (PAS) and methyl green pyronin (MGP). Acid phosphatase (AP) was demonstrated as described by Li *et al.* (1970); nonspecific (napthyl acetate) esterase (NSE) and chloroacetate esterase by the method of Yam *et al.* (1971).

*Histology.*—Material for paraffin and methacrylate embedding was fixed in 10% formol saline. Sections were stained with haematoxylin and eosin, Giemsa, PAS, MGP and for reticulin (Gordon & Sweet), and also stained for cytoplasmic immunoglobulin (light and heavy chains) and lysozyme using an immunoperoxidase technique (Burns, 1975).

TABLE I.—*Clinical features of* 6 *patients with T-cell and* 1 *with B-cell lymphoma*

| Case No. | Age (yrs) | Sex | Mediastinal involvement | Marrow involvement (trephine) | Leukaemia | Other sites involved |
|---|---|---|---|---|---|---|
| 1 | 17 | M | + | – | – | Cervical lymphadenopathy<br>Pleural effusion<br>Hepatomegaly |
| 2 | 18 | M | + | – | – | Supraclavicular and axillary lymphadenopathy<br>Pleural effusion<br>Splenomegaly |
| 3 | 36 | M | + | – | – | Cervical lymphadenopathy<br>Pleural and pericardial effusions<br>Hepatomegaly |
| 4 | 2 | F | – | –<br>(aspirate) | – | Cervical lymphadenopathy<br>Skin nodules<br>Hepatomegaly |
| 5 | 6 | M | – | – | – | Cervical lymphadenopathy |
| 6 | 8 | F | + | + | + | Cervical and supraclavicular lymphadenopathy<br>Pleural and pericardial effusions |
| 7* | 30 | M | + | + | + | Cervical and supraclavicular lymphadenopathy |

* Poorly differentiated lymphocytic lymphoma (B cell).

FIG. 1.—Case 1, showing histiocytes containing phagocytosed material interspersed amongst large lymphoid cells. × 320 H.&E.

FIG. 2.—Case 2, showing a predominance of cells with rounded nuclei and only occasional large cells with convoluted nuclei (arrows). Methacrylate. × 500 Solochrome Cyanin.

FIG. 3.—Case 3, showing a predominance of large cells with convoluted nuclei and nucleoli. Methacrylate. × 500 Solochrome Cyanin.

FIG. 4.—Case 7, showing large cells with cleaved (C) and non-cleaved (N) nuclei. Methacrylate. × 500 Solochrome Cyanin.

Methacrylate sections were stained with solochrome cyanine (Hogg & Simpson, 1975).

### RESULTS

The main clinical features are summarized in Table I. Cases 1–5 are cases of T-cell lymphoma without marrow involvement. For comparison, Case 6 is a case of typical childhood lymphoblastic lymphoma-leukaemia of T-cell type, whilst Case 7 is a B-cell poorly differentiated lymphocytic lymphoma with a leukaemic phase.

### *Morphological studies*

On Rappaport's classification, Cases 1, 2, 4, 5, 6 and 7 were diffuse, poorly differentiated lymphocytic lymphomas, while Case 3 was diffuse, histiocytic lymphoma. All cases showed diffuse infiltration of lymph-node architecture and extension of tumour into perinodal fat. In Case 5 a second lymph-node biopsy showed localization of tumour infiltrate in the paracortical regions. The mitotic rate was always high.

Some T-cell lymphomas showed a prominent starry-sky appearance, with reactive histiocytes interspersed amongst neoplastic cells (Fig. 1). Numbers of convoluted cells varied from case to case and were difficult to identify in paraffin sections. In 1–2$\mu$m thick methacrylate sections these were more easily seen (Fig. 2). In Case 3 the tumour was composed of large cells with predominantly convoluted nuclei and prominent nuclei (Fig. 3).

Special stains on formalin-fixed paraffin-embedded tissue showed strong cytoplasmic pyroninophilia, but immunoperoxidase-stained sections were negative for intracellular immunoglobulin and lysozyme.

Lymph-node imprints stained with May-Grunwald Giemsa showed similar cytological features in all cases examined (2, 4, 5 and 6). Sheets of large lymphoid cells with finely dispersed chromatin and round nuclei without obvious nucleoli were seen. Occasional large blast cells surrounded by a rim of deeply basophilic cytoplasm were also present. In cytocentrifuge preparations of lymph-node cell suspensions, cells with convoluted nuclei could be found in all cases, though these were not obvious in sections or imprints.

In the B-cell lymphoma (Case 7) paraffin sections showed a diffuse infiltration of lymph node by large mononuclear cells with both cleaved and non-cleaved nuclei, the latter containing prominent nucleoli and with moderate surrounding cytoplasm (Fig. 4). There was extensive capsular and perinodal infiltration, and a high mitotic rate. Imprints showed large lymphoid cells 10–20 $\mu$m in diameter with nuclei containing 1–2 prominent nucleoli and with variable amounts of surrounding cytoplasm. MGP-stained sections and imprints showed strongly positive cytoplasm. Immunoperoxidase-stained sections showed a small number of cells containing cytoplasmic immunoglobulin.

In Cases 1, 2, 3 and 7 examination of pleural-fluid smears and cytocentrifuge preparations showed a mixed population of small lymphocytes and large neoplastic lymphoid cells with frequent mitoses. Macrophages, serosal cells and a small number of neutrophils were also present.

### *Cytochemistry*

The cytochemical findings on lymph-node imprints are shown in Table II. In all cases diagnosed as T-cell lymphoma, strongly positive single-spot acid phosphatase (SSAP) was evident in 80–90% of the cells (Fig. 5). In one case (5) cells also showed single-spot NSE activity in over 90% of cells. A smaller number of single-spot NSE$^+$ cells were present in other cases. The neoplastic cells in all T-cell lymphomas unexpectedly showed fine granular cytoplasmic staining for chloroacetate esterase that was easily distinguishable from the strong staining in polymorphonuclear leucocytes. In Case 7 (B-cell) cytochemical stains were negative for acid phosphatase, nonspecific esterase and chloroacetate esterase.

TABLE II.—*Immunological and cytochemical markers in lymph nodes*

| Case No. | % E | % Surface Ig | % Phagocytic cells | Acid phosphatase | Non-specific esterase |
|---|---|---|---|---|---|
| 2 | 35 | 12 | 1·5 | + + | + |
| 4 | 71 | 7 | 1·0 | + + | + |
| 5a | 67 | 11 | 1·0 | + | + + |
| 5b | 37 | 8 | 1·0 | + | + + |
| 6 | 51·5 | 8·5 | 2·0 | + + | + |

+ +, 50–100% cells positive.

+, 10–50% cells positive.

*Immunological studies*

The immunological findings in lymph-node cell suspensions from 4 of the cases diagnosed histologically as T-cell lymphoma are summarized in Table II. The percentage of E-rosetting cells varied. In cases with low percentages there was a large receptor-silent population (*i.e.* cells not forming E rosettes, or with surface Ig). In all cases few cells with detectable immunoglobulin were found. In Cases 1 and 3 fresh tissue was not available.

Cytocentrifuge preparations of rosetting cells from these 4 cases showed medium and large lymphoblasts forming E rosettes. These cells did not form rosettes with IgG or complement-coated ox RBC.

Immunological studies on pleural fluids in Cases 1, 2 and 3 showed a high proportion of E-rosetting cells (Table III). These were large lymphoblastic cells, some with convoluted nuclei (Fig. 6). Variable numbers of cells with surface Ig were also present. These were small cells with capping surface Ig and with a polyclonal pattern of light-chain expression.

In Case 7 the E-rosetting cells were small, morphologically normal cells (Fig. 6). The large lymphoblastic cells present did not form E rosettes, but had monoclonal surface Ig (IgM $\lambda$) and intracytoplasmic Ig.

TABLE III.—*Immunological markers on pleural-fluid cells*

| Case No. | Lymphoma type | E rosettes (%) | Surface Ig (%) | $\kappa$ Chain (%) | $\lambda$ Chain (%) | SSAP* |
|---|---|---|---|---|---|---|
| 1 | T | 67·5 | 45 | ND | ND | + + + |
| 2 | T | 83 | 6 | 4 | 5 | + |
| 3 | T | 58 | 18·5 | 12 | 20 | + |
| 7 | B | 56 | 57 | 0 | 29 | + |

* SSAP—Single-spot acid-phosphatase activity.

FIG. 5.—Case 2, lymph-node imprint showing strong focal acid-phosphatase activity (arrow) in lymphoid cells. × 1250.

FIG. 6.—E rosetting cells in pleural fluid. Large convoluted cells in Cases 1, 2 and 3. Small cell in Case 7. × 1250 May–Grunwald Giemsa.

TABLE IV.—*Chemotherapy and response to treatment*

| | | | | Duration of | |
|---|---|---|---|---|---|
| Case No. | Chemo-therapy | CNS Prophyl-axis | Response | Re-mission (mths) | Survival (mths) |
| 1 | CHOP | + | CR | 8* | 18+ |
| | CHOP | | CR | 10 | |
| | | (2nd remission) | | | |
| 2 | APV | + | CR | 9 | 15+ |
| 3 | APV | – | PR | 2† | 13 |
| 4 | CHOP | + | CR | 22 | 28+ |
| 5 | CHOP | + | CR | 18‡ | 28+ |
| | CHOP | | CR | 10 | |
| 6 | —§ | – | — | — | 0 |
| 7 | MVPP | – | IF | 0 | 7 |

* Relapse after 8 months with acute leukaemia.

† Chemotherapy changed to COAP after 2 months because of development of pulmonary infiltration. Died after 13 months with disseminated disease despite intensive chemotherapy.

‡ Relapse with recurrent cervical adenopathy.

§ Died before chemotherapy commenced.

CR = complete remission; PR = partial remission; IF = induction failure; CHOP = cyclophosphamide, adriamycin, vincristine, prednisolone; COAP = cyclophosphamide, vincristine, cytosine arabinoside, prednisolone; APV = adriamycin, prednisolone, vincristine; MVPP = mustine, vinblastine, prednisolone, procarbazine.

At presentation patients had normal white-cell counts, except for Cases 6 and 7, which were leukaemic. In Cases 1–6 immunological studies on peripheral-blood mononuclear cells showed that the percentage of E rosettes varied widely between cases but was usually normal or high. Peripheral-blood lymphocytes of all untreated patients with T-cell lymphoma formed a higher proportion of heat-stable E rosettes in normal individuals. The percentages of cells with surface Ig were normal or low, with normal distribution of $\kappa$ and $\lambda$ light chains. In Cases 3, 4 and 6 there were large receptor-silent cell populations. In Case 6 large numbers of blasts were present in blood. All the blasts showed single-spot acid phosphatase activity. Some blasts, including typical convoluted cells, formed E rosettes. In Case 1 relapse occurred 8 months after diagnosis. In peripheral blood there were convoluted blast cells forming E rosettes, over 90% of which were SSAP$^+$.

In Case 7 large numbers of blasts with non-convoluted nuclei were present in blood. These cells expressed monoclonal surface IgM $\lambda$ and did not form E rosettes.

*Response to treatment*

The treatment schedules are summarized in Table IV. Patients 1–5 were treated with the combined chemotherapy regime including adriamycin, prednisolone and vincristine. In addition, cyclophosphamide was given to Cases 1, 4 and 5. CNS prophylaxis was by intrathecal methotrexate and cranial irradiation. The B-cell lymphoma (Case 7) was treated with mustine, vinblastine, prednisolone and procarbazine (MVPP).

### DISCUSSION

In this paper we describe several cases of T-cell lymphoma, only one of which (Case 6) could be recognized by characteristic histological and clinical features of a T-cell lymphoma: mediastinal mass, marrow infiltration and convoluted-cell leukaemia. The two other paediatric cases presented with asymptomatic cervical adenopathy (Cases 4 and 5) and skin nodules (Case 4) with no evidence of marrow involvement or mediastinal tumour. On histological criteria, Cases 4 and 5 were classified simply as diffuse poorly differentiated lymphocytic lymphomas.

In our 3 adult cases, the first 2 were also initially diagnosed as poorly differentiated lymphocytic lymphoma, and Case 3 as histiocytic lymphoma. Although all 3 had clinical features suggestive of the diagnosis of T-cell lymphoma, none had marrow involvement at diagnosis.

These cases illustrate the heterogeneity of morphology in the T-cell lymphomas (Habeshaw *et al.*, 1979). Cases 1, 2, 4, 5 and 6 are of the convoluted, or lymphoblastic, cell type of lymphoblastic lymphoma, whereas Case 3 more closely resembles the large-cell type of T-cell lymphoma described by Mann (1975) and Waldron *et al.* (1977).

In our 3 adult cases, despite the differences in histological appearance, they nevertheless appear to form a clinically distinct group of lymphoma with predilection for mediastinal and supradiaphragmatic lymph nodes and pleural involvement.

Difficulties in basing the diagnosis on morphological and clinical criteria alone are illustrated by Case 7, in which a young man with mediastinal and supraclavicular lymph adenopathy, pleural effusions, marrow infiltration and leukaemia, was shown to have a neoplasm that was clearly of B-cell origin. Histology in this case showed a diffuse lymphocytic lymphoma composed of cells with irregular nuclei, which were interpreted as being cleaved B cells rather than convoluted T cells only after immunological studies on blood and pleural fluid had shown neoplastic cells expressing monoclonal surface Ig.

The T-cell origin of the neoplastic cells in Cases 1–6 was confirmed by finding neoplastic cells expressing receptors for sheep red cells either in lymph nodes or pleural fluid. In cell suspensions precise identification of cells was difficult and cytospin preparations stained with Giemsa were necessary reliably to identify neoplastic cells with receptors for sheep RBC. Similarly in pleural fluid in Cases 1, 2 and 3, cytospin preparations were necessary to identify rosetting neoplastic cells and distinguish these from other cells. Further evidence for the T-cell nature of these cases was given by the strong focal or single spot acid-phosphatase activity of cells in lymph-node imprints (Catovsky & Enno, 1977). In Case 2 Tdt (terminal deoxynucleotidyl transferase) levels in lymph nodes and pleural fluid were high, consistent with T-cell origin (Bollum, 1979). No material from other cases was available for Tdt estimation.

Our findings show how a combination of clinical, pathological and immunological techniques are necessary for precise evaluation of patients with malignant lymphoma. This is especially true in younger patients who do not present with typical clinical features and where identification of the neoplastic cell type may have important therapeutic and prognostic implications (Schneider *et al.*, 1975; Bloomfield *et al.*, 1977; Rosen *et al.*, 1978; Weinstein *et al.*, 1979).

The importance of recognizing T-cell lymphoma in the absence of leukaemia or overt marrow involvement has recently been emphasized by the finding that an aggressive approach to therapy induction and maintenance can achieve remission in most of these patients (Hausner, 1977). Previous reports of treatment of T-cell lymphoma by conventional chemotherapy have shown a poor response, with rapidly developing resistance to therapeutic agents. However, using combination chemotherapy regimes that are effective in acute lymphocytic leukaemia, more encouraging results have been obtained (Weinstein *et al.*, 1979). Patients with leukaemia or marrow infiltration at diagnosis still have a poor prognosis (Catovsky *et al.*, 1974). Four of the 5 patients who presented with no marrow involvement are still alive at the time of writing.

We are grateful to Drs O. B. Eden, J. Syme, S. H. Davies and A. C. Parker for the clinical information about these patients. The work was done under the auspices of the Edinburgh Lymphoma Group in the Department of Pathology, University of Edinburgh. One of us (A.S.K.) holds an M.R.C. Junior Research Fellowship. The authors are indebted to Miss E. F. Ramage and Miss J. Kidby for their expert technical assistance.

### REFERENCES

BLOOMFIELD, C. D., KERSEY, J. H., BRUNNING, R. D. & GAJL-PECZALSKA, K. J. (1977) Prognostic significance of lymphocytic surface markers and histology in adult non-Hodgkin's lymphoma. *Cancer Treatment Rep.*, **61**, 963.

BOLLUM, F. J. (1979) Terminal deoxynucleotidyl transferase as a haematopoietic cell marker. *Blood*, **54**, 1203.

BURNS, J. (1975) Background staining and sensitivity of the unlabelled antibody-enzyme (PAP) method. Comparison with peroxidase labelled antibody sandwich method using formalin fixed paraffin embedded material. *Histochemistry*, **42**, 291.

CATOVSKY, D. & ENNO, A. (1977) Morphological and cytochemical identification of lymphoid cells. *Lymphology*, **10**, 77.

CATOVSKY, D., PETTET, J. E., GALETTO, J., OKOS, A. & GALTON, D. A. G. (1974) The B lymphocyte nature of the hairy cell of leukaemic reticuloendotheliosis. *Br. J. Haematol.*, **26**, 29.

HABESHAW, J. A., CATLEY, P. F., STANFELD, A. G. & BREARLEY, R. L. (1979) Surface phenotyping, histology and the nature of non-Hodgkin's lymphoma in 157 patients. *Br. J. Cancer*, **40**, 11.

HABESHAW, J. A. & STUART, A. E. (1975) Cell receptor studies on seven cases of diffuse histiocytic malignant lymphoma (reticulum cell sarcoma). *J. Clin. Path.*, **28**, 289.

HABESHAW, J. A. & YOUNG, G. A. (1975) Quantitation of sub-classes of mononuclear cells in normal human blood by membrane receptor studies. *Br. J. Haematol.*, **29**, 43.

HAUSNER, R. J., ROSAS-URIBE, A., WICKSTRUM, D. A. & SMITH, P. C. (1977) Non Hodgkin's lymphoma in the first two decades of life. *Cancer*, **40**, 1533.

HOGG, R. M. & SIMPSON, R. (1975) An evaluation of solochrome cyanine RS as a nuclear stain similar to haematoxylin. *Med. Lab. Technol.*, **32**, 301.

JAFFE, E. S., BRAYLAN, R. C., NANBA, K., FRANK, M. J. & BERARD, C. W. (1977) Functional markers: A new perspective on malignant lymphomas. *Cancer Treatment Rep.*, **61**, 953.

LENNERT, K., MOHRI, N., STEIN, H. & KAISERLING, E. (1975) The histopathology of malignant lymphoma. *Br. J. Haematol.*, **31**, 193.

LI, C. Y., YAM, L. T. & LAM, K. W. (1970) Studies of acid phosphatase isoenzymes in human leukocytes demonstration of isoenzyme cell specificity. *J. Histochem. Cytochem.*, **18**, 901.

LUKES, R. J. & COLLINS, R. D. (1975) New approaches to the classification of the lymphomata. *Br. J. Cancer*, **31**, 1.

LUKES, R. J., TAYLOR, C. R., PARKER, J. W., LINCOLN, T. L., PATTENGALE, P. K. & TINDLE, B. H. (1978) A morphologic and immunologic surface marker study of 299 cases of non-Hodgkin lymphomas and related leukaemias. *Am. J. Pathol.*, **90**, 461.

MANN, R. B., JAFFE, E. S., BRAYLAN, R. C. & 4 others (1975) Immunologic and morphologic studies of T cell lymphoma. *Am. J. Med.*, **58**, 307.

NATHWANI, B. N., KIM, H. & RAPPAPORT, H. (1976) Malignant lymphoma, lymphoblastic. *Cancer*, **38**, 964.

PALUTKE, M., PATT, D. J., WEISE, R. & 4 others (1977) T cell leukaemia–lymphoma in young adults. *Am. J. Clin. Pathol.*, **68**, 429.

PINKUS, G. S. & SAID, J. W. (1979) Characterization of non Hodgkin's lymphomas using multiple cell markers: immunologic, morphologic and cytochemical studies of 72 cases. *Am. J. Pathol.*, **94**, 349.

RAPPAPORT, H. (1966) Tumours of the haemtopoietic system. In *Atlas of Tumour Pathology, Section III*, Fascide 8, Washington, D.C.: Armed Forces Institute of Pathology, p. 97.

ROSEN, P. J., FEINSTEIN, D. T., PATTENGALE, P. K. & 6 others (1978) Convoluted lymphocytic lymphoma in adults. A clinicopathologic entity. *Ann. Int. Med.*, **89**, 319.

SCHNEIDER, B. K., HIGGINS, G. R., SWANSON, V., ISSACS, H., TINDLE, S. H. & LUKES, R. J. (1975) Malignant lymphomas of childhood. *Blood*, **46**, 1015.

SIMS, B. (1974) A simple method of preparing 1–2$\mu$m sections of large tissue blocks using glycol methacrylate. *J. Microscopy*, **101**, 223.

WALDRON, J. A., LEECH, J. H., GLICK, A. D., FLEXNER, J. M. & COLLINS, R. D. (1977) Malignant lymphoma of peripheral T-lymphocyte origin. *Cancer*, **40**, 1604.

WEINSTEIN, H. J., VANCE, Z. B., JAFFE, N., BUELL, D., CASSADY, J. R. & NATHAN, D. G. (1979) Improved prognosis for patients with mediastinal lymphoblastic lymphoma. *Blood*, **53**, 687.

WILLIAMS, A. H., TAYLOR, C. R., HIGGINS, G. R. & 10 others (1978) Childhood lymphoma–leukaemia I. Correlation of morphology and immunological studies. *Cancer*, **42**, 171.

YAM, L. T., LI, C. Y. & CROSBY, W. H. (1971) Cytochemical identification of monocytes and granulocytes. *Am. J. Clin. Pathol.*, **55**, 283.